# Multicenter evaluation of the eQUANT system for use with disk diffusion AST of gram-negative bacteria directly from positive blood cultures

**DOI:** 10.1128/jcm.01606-24

**Published:** 2025-05-09

**Authors:** Sanjucta Dutta, Michael R. Jacobs, Caryn E. Good, Ayman M. Abdelhamed, Yun C. Ying, Adrian Hoyos-Urias, Derrick J. Sugui, Jacqueline U. Anaeto, Eszter Deak, Rachael Valdez, Nitin K. Rajan, Meike S. Herget, Stefan Riedel

**Affiliations:** 1 Department of Pathology, Beth Israel Deaconess Medical Center 1859 , Boston, Massachusetts, USA; 2 Harvard Medical School 1811 , Boston, Massachusetts, USA; 3 University Hospital Cleveland Medical Center and Case Western Reserve University 24575, Cleveland, Ohio, USA; 4 Quest Diagnostics, Lewisville, Texas, USA; 5 Avails Medical, Inc., Menlo Park, California, USA; Johns Hopkins University, Baltimore, Maryland, USA

**Keywords:** bloodstream infections (BSIs), sepsis, eQUANT system, eMcFarland, standardized inoculum, positive blood cultures (PBCs), antimicrobial susceptibility testing, disk diffusion testing

## Abstract

Bloodstream infections frequently cause sepsis, a condition that can lead to organ dysfunction and death. Rapid antimicrobial susceptibility testing (AST) results are critical for appropriate medical intervention and to either de-escalate or escalate antibiotics to appropriate therapy. We describe the results from a multicenter clinical study evaluating the performance of the eQUANT system (Avails Medical, Inc., Menlo Park, CA). The eQUANT system generates a 0.5 McFarland equivalent suspension, the eMcFarland, directly from a positive blood culture bottle for use with downstream disk diffusion AST, thereby saving up to 24 hours compared to traditional AST workflow. A combination of fresh, prospectively collected clinical (42) and contrived (525) blood cultures were tested, and results for disk diffusion using the Avails eQUANT eMcFarland suspension as the inoculum were compared to disk diffusion results the next day using the standard 0.5 McFarland suspension prepared from plate subculture. Thirteen species of gram-negative rods were evaluated against 12 antibiotics. From the 2,679 pairs of AST results, overall categorical agreement was 95.0%. Overall very major errors, major errors, and minor errors were 0.15%, 0.60%, and 4.48%, respectively. The Avails eQUANT system has the potential to significantly accelerate the standard of care by eliminating the need for subculture.

**IMPORTANCE:**

Rapid reporting of antimicrobial susceptibility test results for bacterial isolates from blood cultures is critical for timely implementation of optimal antimicrobial therapy and improves outcomes of sepsis patients. In this study, we demonstrate the accuracy and performance characteristics of the eQUANT system, which generates a 0.5 McFarland equivalent suspension, the eMcFarland, directly from a positive blood culture bottle for use with downstream disk diffusion antimicrobial susceptibility testing (AST). Using the eMcFarland suspension allows for accurate and standardized reporting of disk diffusion AST results from positive blood cultures.

## INTRODUCTION

Bloodstream infections and sepsis are major causes of morbidity and mortality worldwide (1, 2). In the USA, every year, at least 1.7 million adults develop sepsis, with at least 350,000 deaths annually (3). In the USA, sepsis is the most common cause of in-hospital deaths, and associated costs exceed $24 billion annually (3–5). Blood culture (BC) remains the most important cornerstone of sepsis diagnosis, considering that the identification of the offending bacterial or fungal organism is essential to selecting optimal antimicrobial therapy (6–8). Improvements to continuously monitored BC systems and BC broths during the past decades have improved the ability to recover the causative microorganisms. Antimicrobial susceptibility testing (AST) of blood culture isolates is essential to optimize the patient’s antimicrobial therapy, enabling either de-escalation or escalation of antibiotics to more appropriate or optimal therapy (9, 10). While most microbiology laboratories can use technologies for faster organism identification from positive BCs, traditional AST methods still require between 24 and 96 hours. AST is traditionally performed by broth microdilution (BMD) testing or a BMD-derived method, including automated AST systems or disk diffusion testing. However, BMD and disk diffusion testing have a few technical limitations, in addition to the longer turnaround times (TATs) of results reporting. Such limitations include the need for a bacterium to grow in pure culture on agar plates inoculated from positive BC bottles and the need for a standardized inoculum of the bacterium, measured by a standardized McFarland suspension. The latter is a manual process and fraught with some variability in obtaining an accurate and standardized suspension, partly due to a mix of viable and dead organisms. Despite these limitations, BMD is still considered the gold standard, as AST results have repeatedly been shown to be most predictive of treatment outcome in sepsis patients (11). In addition, modifications of the disk diffusion (DD) testing method have been developed for rapid AST directly from positive blood cultures; such methods have subsequently been approved by the Clinical and Laboratory Standards Institute (CLSI) and the European Committee on Antimicrobial Susceptibility Testing (EUCAST) (12, 13). These methods, however, require use of non-standardized inocula, adjustments to laboratory workflow, and use of alternate breakpoints, compared to standard DD methods. There are a few rapid genotypic diagnostic test methods that provide not only organism identification (ID) directly from a positive blood culture (PBC), but also the detection of select antimicrobial resistance genes (14). Results with these instruments, e.g., BioFire (bioMérieux), Verigene (Diasorin), ePlex (Roche Diagnostics), are available within 1.5 to 4 hours after a BC bottle turns positive and a Gram stain is performed. However, these genotypic detection methods can only be considered to be supplemental to the actual determination of full range, phenotypic antimicrobial susceptibility testing, since only a limited set of antimicrobial resistance genes for gram-positive and/or gram-negative bacteria is included on these panels. Therefore, it is desirable to expedite and shorten the time of the procedural steps to perform full-scale phenotypic AST following standard laboratory practices.

In this regard, the eQUANT instrument (Avails Medical, Inc.) is a small (21.6 cm × 11.7 cm × 21.6 cm) bench-top automated instrument which uses potentiometric sensing of changes in oxidation-reduction potential (ORP) from gram-negative or gram-positive pathogen metabolism in cation-adjusted Mueller-Hinton broth (CAMHB) with an antifoaming agent to prepare an organism suspension (eMcFarland) equivalent to a traditional 0.5 McFarland (1–2 × 10^8^ CFU/mL ± 0.6 log or 2.51 × 10^7^ to 7.96 × 10^8^ CFU/mL; CFU: colony-forming unit) directly from PBC broth in 64–72 minutes on average (15, 16). For each organism and blood culture bottle type combination, there exists a mapping of changes in ORP which correlates to changes in the bacterial cell density (15). Thus, by monitoring the ORP changes in real-time, the system can precisely determine the time at which the bacterial cell density reaches the desired count (equivalent to a 0.5 McFarland). Based on the user selections of organism and bottle type, the appropriate algorithm (and algorithm parameters) is applied, and as soon as the desired cell density is reached, the system rapidly cools down the sample to 15°C to limit further growth and starts a countdown timer for 1 hour (15). This eMcFarland suspension of the respective organism can then be used to set up the traditional phenotypic AST using methods available to the respective clinical laboratory. This approach eliminates the need to grow the organism in pure culture, therefore shortening the overall TAT from the time of a positive BC bottle to the final AST result by up to 24 hours. Currently, the eQUANT system is FDA cleared for use with a panel of gram-negative organisms with disk diffusion AST (17). Here, we report the results from the evaluation of the Avails Medical eQUANT System for gram-negative AST using the disk diffusion method at three clinical microbiology laboratories, using prospective clinical blood culture samples positive for gram-negative rods, as well as contrived blood culture samples . The AST results using the eQUANT eMcFarland standard were compared to the AST results obtained the next day using the currently established standard method for disk diffusion AST with the manually prepared McFarland standard from plate subculture.

## MATERIALS AND METHODS

### Study sites

This study was performed at three clinical microbiology laboratories, each affiliated with an academic medical center, as well as at Avails Medical, Inc. in Menlo Park, CA; the participating laboratories included Beth Israel Deaconess Medical Center (BIDMC), Boston, MA, University Hospitals Cleveland Medical Center (UHCMC), Cleveland, OH, and Quest Diagnostics (Quest) in Lewisville, TX. All discrepant result testing was performed at Avails Medical, Inc.

### Study design

**Fig 1:**
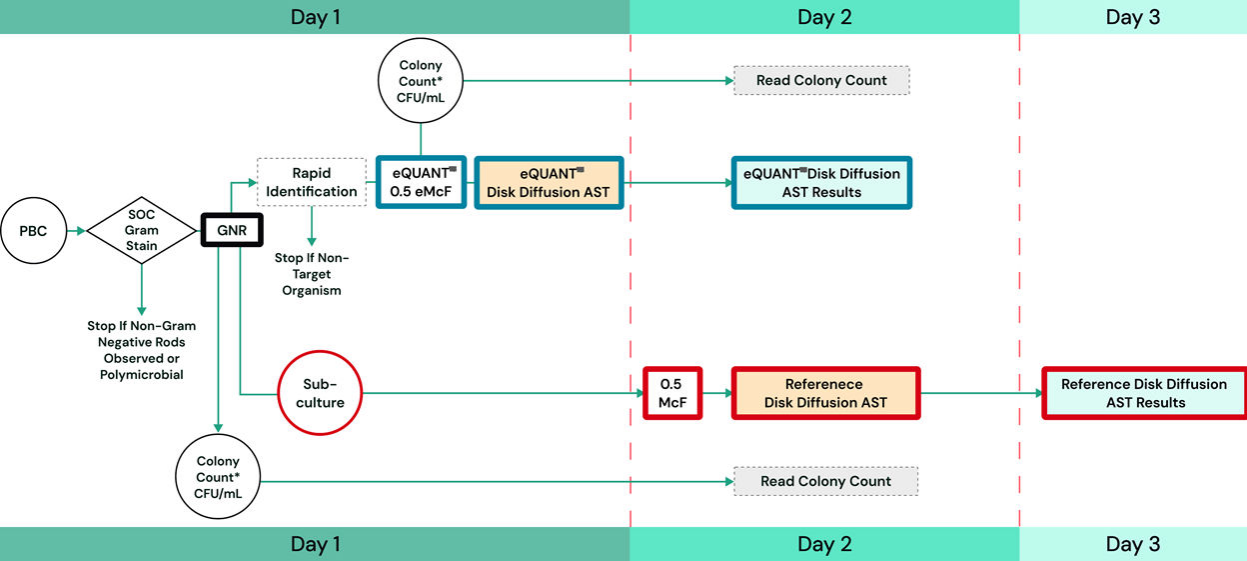
Clinical workflow. The study was designed to compare disk diffusion AST results obtained with the eQUANT eMcFarland to results obtained with a standard 0.5 McFarland from subculture. The workflow started on day 1 with a PBC that was analyzed by standard of care (SOC) Gram stain for gram-negative rods. If gram-negative, a rapid identification was performed using a direct rapid ID method. If the identified organism covered an organism that is intended for use with the eQUANT system, the sample was subcultured and incubated overnight, and at the same time, it was processed on the eQUANT system for McFarland generation. The eMcFarland was subsequently plated for colony counts and processed for disk diffusion AST. On day 2, eMcFarland colony counts were read, as well as (eQUANT) disk diffusion results. Additionally, reference disk diffusion AST was set up by preparing a 0.5 McFarland from the colonies of the subcultured PBC. On day 3, reference disk diffusion AST results were read and compared to results obtained with the eQUANT eMcFarland.

All testing was performed on prospectively collected, de-identified positive BC bottles routinely processed by the clinical sites after clinical testing was completed or on positive blood cultures contrived with stock or challenge gram-negative isolates. Prospective samples were enrolled if they met the following requirements: (i) flagged positive by the site’s continuous monitoring blood culture system, (ii) confirmed to contain gram-negative rods via Gram stain, (iii) identified to be positive for target gram-negative organisms by an FDA-cleared rapid ID system, and (iv) within 12 hours of the blood culture flagging positive. The study had two objectives: (i) to verify that the count of the eQUANT eMcFarland output suspension was between 2.51 × 10^7^ and 7.96 × 10^8^ CFU/mL by quantitative plate count, i.e., to be equivalent to a traditional 0.5 McFarland suspension; and (ii) to compare the disk diffusion AST results obtained with the eMcFarland inoculum to the results obtained with a traditional 0.5 McFarland suspension prepared from colonies on a subculture plate the next day (Fig. 1).

### Clinical specimens

At each clinical site, prospective positive BCs were screened using Gram stain results to select for monomicrobial cultures with the presence of a gram-negative rod (GNR). Each laboratory then performed a rapid organism identification with the respective system validated and in use in their clinical laboratory. The laboratory technologies used for blood culture incubation and organism identification from BCs positive for GNRs are listed in Table 1. When identified by these rapid ID systems, the following bacterial species were included for disk diffusion AST using the eQUANT system in parallel to performing the standard disk diffusion method the next day with a traditional 0.5 McFarland suspension prepared from plate subcultures: *Acinetobacter* spp., *Citrobacter* spp., *Enterobacter* spp., *Escherichia coli, Klebsiella aerogenes, Klebsiella pneumoniae, Klebsiella oxytoca, Proteus* spp., *Pseudomonas aeruginosa,* and *Serratia marcescens* (Table 2). Samples with mixed Gram stain results or samples with multiple bacterial organisms being isolated and identified in the same BC bottle were excluded from the study. In addition, contrived blood cultures were prepared and tested using stock isolates (provided by the clinical site and/or by Avails Medical) or challenge isolates with known resistance profiles (provided by Avails Medical and previously obtained from the CDC Antimicrobial Resistance Isolate Bank) (18). Blood culture bottles were inoculated with 6 mL–9 mL of human donor whole blood, containing sodium citrate (Lampire Biological Laboratories), and the respective bacterial stock isolate or challenge isolate was added. Contrived specimens were used to supplement less frequently isolated species and resistance profiles, as well as to supplement overall sample numbers. The contrived specimen included strains of species such as *Citrobacter freundii, Escherichia coli, Klebsiella oxytoca*, *Klebsiella pneumoniae*, and *Serratia marcescens* with characterized resistance mechanisms such as extended-spectrum β-lactamases (ESBLs) and carbapenemases. Of the samples tested in the study, 95 had genotypically confirmed resistance markers, including 9 *Acinetobacter* spp., 2 *Citrobacter freundii*, 12 *Enterobacter cloacae*, 37 *Escherichia coli*, 4 *Klebsiella aerogenes*, 12 *Klebsiella pneumoniae*, 13 *Pseudomonas aeruginosa*, 3 *Proteus mirabilis*, and 3 *Serratia marcescens* isolates (Table S1). Additionally, there were 30 isolates that were identified phenotypically as ESBL-positive using MicroScan Negative MIC 43 panels, including 16 *Escherichia coli*, 4 *Klebsiella oxytoca*, 5 *Klebsiella pneumoniae*, and 5 *Proteus mirabilis*. Contrived BCs were incubated in the continuous monitoring blood culture instrument used at the respective participating institution (Table 1). Positive BC samples were processed on the eQUANT instrument as described below in eQUANT testing within 12 hours of the blood culture flagging positive. At all participating laboratory sites, the eMcFarland standard was obtained by using the eQUANT Instrument; all disk diffusion AST was performed following current CLSI guidelines and interpreted following FDA guidelines (19–21).

**TABLE 1:** Blood culture systems and systems used for organism identification, by study site

Laboratory site	BIDMC	UHCMC	Quest	Avails
Blood Culture System	BACTEC FX	BacT/ALERT VIRTUO	BacT/ALERT 3D and BacT/ALERT VIRTUO	BACTEC FX and BacT/ALERT 3D
Rapid ID System	BioFire	Verigene	Verigene	N/A^*a*^
Number of Prospective samples enrolled	13	15	14	0
Number of contrived samples enrolled	44	47	22	412

^*a*^ N/A: not applicable.

**TABLE 2:** Overview of tested organisms in the clinical study

Organism details	Prospective	Contrived	Total	Percent
*Acinetobacter* species	0	41	41	7.23%
*Citrobacter freundii*	1	40	41	7.23%
*Citrobacter* species	0	2	2	0.35%
*Enterobacter cloacae*	0	45	45	7.94%
*Escherichia coli*	25	157	182	32.10%
*Klebsiella aerogenes*	1	42	43	7.58%
*Klebsiella oxytoca*	0	19	19	3.35%
*Klebsiella pneumoniae*	11	30	41	7.23%
*Proteus mirabilis*	1	51	52	9.17%
*Proteus spp*.	1	0	1	0.18%
*Proteus vulgaris*	0	18	18	3.17%
*Serratia marcescens*	1	40	41	7.23%
*Pseudomonas aeruginosa*	1	40	41	7.23%
*Total*	42	525	567	100.00%

### eQUANT testing

For sample processing on the eQUANT instrument, 1 mL of the eQUANT reagent (CAMHB with 0.0015% antifoam) was transferred into a disposable eTube. The eTube was then inoculated with 34 µL of PBC and run on the eQUANT instrument as per the manufacturer’s instructions for use. After run completion, the ensuing eMcFarland suspensions were allowed to remain in the eQUANT instrument for up to 1 hour at 15°C and processed for colony counts and disk diffusion AST within 10 minutes of removal from the instrument. For comparator method testing, the positive BCs were then subcultured onto sheep blood agar plates and incubated overnight for next day preparation of a traditional 0.5 McFarland suspension for use with disk diffusion AST.

### Quantitation of inocula

Quantitative cultures were performed on PBC and eMcFarland samples by the drop count method, with PBC samples serially diluted 1:10^8^ in four steps and eMcFarland samples 1:10^7^ in three steps. The final dilutions were cultured by plating seven 10 µL volumes on blood agar plates, which were incubated at 35°C overnight. The average number of colonies per 10 µL spot was multiplied by the dilution factor to obtain the colony count of the specimen in CFU/mL.

### Disk diffusion testing

**Fig 2:**
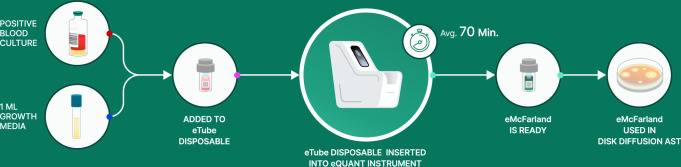
eQUANT sample preparation workflow. To prepare a sample for run on the eQUANT system, 34 µL positive blood culture is combined with 1 mL of eQUANT reagent (CAMHB with 0.0015% antifoam) into the eTube disposable. The eTube with diluted PBC sample is then inserted into the eQUANT instrument. Upon selection of the organism ID and blood culture bottle type, the run is started, and the eMcFarland is generated in an average of 64 minutes. Once the run is complete, the eMcFarland is removed and used for disk diffusion AST. Alternatively, it can be stored on the instrument for up to 1 hour where it gets cooled to 15°C until being further processed in downstream disk diffusion.

Qualitative AST performance for disk diffusion was assessed with 12 antimicrobials (Table 3) performed for all samples at all sites. The corresponding eQUANT workflow and study workflow are shown in Fig. 1 and 2, respectively. Antimicrobial susceptibility testing using the disk diffusion method was performed in accordance with CLSI guidelines for antimicrobial disk diffusion testing (CLSI M02) and FDA disk diffusion AST result interpretations (FDA Interpretive Criteria) (19, 21). Breakpoints used in this study are shown in Table S2, and differences between FDA and CLSI M100 breakpoints are shown in Table S3.

**TABLE 3:** Performance of the eQUANT system used with gram-negative bacteria and disk diffusion testing^*a*^

Antimicrobial agent	Organisms	Number (#) of organisms tested	Categorical interpretation			Categorical Agreement (# and %)	Errors [# and %)		
			S	I	R		VME	ME	mE
Amoxicillin/Clavulanic acid	Enterobacterales	223	150	13	60	210 (94%)	0 (0%)	1 (0.67%)	12 (5.38%
Ampicillin	Enterobacterales	33	9	1	23	33 (100%)	0 (0%)	0 (0%)	0 (0%)
Aztreonam	Enterobacterales	189	141	3	45	183 (97%)	0 (0%)	1 (0.71%)	5 (2.65%)
	Pseudomonas aeruginosa	25	10	4	11	24 (96%)	0 (0%)	0 (0%)	1 (4%)
Cefazolin	Enterobacterales	213	71	44	98	181 (85%)	0 (0%)	1 (1.41%)	31 (14.55%)
Cefepime	Enterobacterales	237	162	33	42	218 (92%)	0 (0%)	1 (0.62%)	18 (7. 59%)
	Pseudomonas aeruginosa	41	21	0	20	39 (95%)	0 (0%)	2 (9.52%)	0 (0%)
Ceftriaxone	Enterobacterales	223	134	5	84	216 (97%)	0 (0%)	2 (1.49%)	5 (2.24%)
Ertapenem	Enterobacterales	187	153	5	29	183 (98%)	0 (0%)	0 (0%)	4 (2.14%)
Gentamicin	Enterobacterales	255	226	3	26	248 (97%)	1 (3.85%)	1 (0.44%)	5 (1.96%)
	Pseudomonas aeruginosa	41	23	1	17	39 (95%)	0 (0%)	0 (0%)	2 (4.88%)
Levofloxacin	Enterobacterales	206	146	11	49	198 (96%)	0 (0%)	0 (0%)	8 (3.88%)
	P. aeruginosa	25	8	6	11	24 (96%)	0 (0%)	0 (0%)	1 (4%)
Meropenem	Acinetobacter spp.	16	6	1	9	16 (100%)	0 (0%)	0 (0%)	0 (0%)
	Enterobacterales	206	180	3	23	200 (97%)	0 (0%)	2 (1.11%)	4 (1.94%)
	Pseudomonas aeruginosa	25	8	0	17	25 (100%)	0 (0%)	0 (0%)	0 (0%)
Piperacillin/Tazobactam	Acinetobacter spp.	41	16	0	25	39 (95%)	0 (0%)	0 (0%)	2 (4.88%)
	Enterobacterales	239	166	27	46	225 (94%)	0 (0%)	0 (0%)	14 (5.86%)
	Pseudomonas aeruginosa	25	14	2	9	24 (96%)	0 (0%)	0 (0%)	1 (4%)
Tobramycin	Enterobacterales	203	164	6	33	196 (97%)	0 (0%)	0 (0%)	7 (3.45%)
	Pseudomonas aeruginosa	25	16	1	8	25 (100%)	0 (0%)	0 (0%)	0 (0%)
TOTAL		2,679	1,825	169	685	2,546 (95%)	1 (0.15%)	11 (0.60%)	120 (4.48%)

^*a*^ VME, very major error; ME, major error; mE, minor error.

Overall disk diffusion performance and disk diffusion performance for each appropriate organism/drug combination were evaluated by comparing results using the eQUANT eMcFarland as the inoculum (test method) to results obtained using a standard 0.5 McFarland prepared from the subculture of the PBC bottle as the inoculum the next day (comparator method). Categorical agreement (CA), very major errors (VMEs), major errors (MEs), and minor errors (mEs) were calculated overall and for each organism/drug combination (17). CA is the percent category interpretation agreement between the test and the comparator results. A VME is the percentage of results for which the comparator is resistant while the test result is susceptible and is calculated by dividing the number of VME discrepancies by the total number of resistant organisms according to the comparator method. An ME is the percentage of results for which the comparator is susceptible while the test result is resistant and is calculated by dividing the number of ME discrepancies by the total number of susceptible isolates according to the comparator method. An mE occurs when the comparator result is intermediate and the test result is either resistant or susceptible, or when the test result is intermediate and the comparator result is either resistant or susceptible. Note that in this study, the results from the eQUANT were compared to standard disk diffusion testing, which is not considered a reference method. FDA drug labeling and FDA breakpoints were used for result interpretation (17). The acceptable performance criteria used for this study, as required by the FDA, were a CA of ≥95%, with VME ≤1% and ME ≤1.5%; no criterion is specified for an acceptable mE rate (17). These criteria were more stringent than those published by the FDA in 2009 (22). A preliminary assessment of VME and ME rates, performed after 136 samples had been tested, showed that denominators of several organism–antimicrobial pairs were low, and supplemental testing of 431 contrived samples was performed at Avails using the relevant antimicrobials needed to obtain meaningful denominators.

For each disk diffusion AST test run and every day of testing with clinical and/or contrived samples, quality control testing was performed using contrived PBCs with one of three Quality Control (QC) organisms in rotation (*E. coli*, ATCC 25922; *K. pneumoniae*, ATCC 700603; *P. aeruginosa*, ATCC 27853), following CLSI guidelines (CLSI M02; CLSI M100) and the manufacturer’s instructions for use (19, 20). QC acceptance criteria were based on eQUANT eMcFarland colony counts and disk diffusion testing results (zone diameters) for the respective QC organisms and antimicrobials tested being within published ranges. The QC pass rate was ≥98% across all sites. Of 20 QC failures observed, 12 were with meropenem and *P. aeruginosa* ATCC 27853. The majority of these failures occurred at one of the sites and were due to a suspected defective lot of meropenem antimicrobial disks at this site. The disks were replaced, and meropenem QC at the site subsequently passed at an acceptable rate. Sample meropenem disk results impacted by the meropenem QC failures were excluded from the performance analysis.

Isolates from samples with VME or ME were sent to Avails Medical, Inc. for discrepant result testing. Isolates were retested as contrived samples using the same blood culture bottle used for that isolate at the clinical site. Contrived blood cultures were incubated until positive and then processed for Standard inoculum and eQUANT eMcFarland disk diffusion testing. Only the organism/drug combination that produced the error was tested. All analyses for this study were performed using the original testing results. Discrepancy testing results were used for informational purposes only to determine the reproducibility of the errors and to determine if discrepancy testing results matched the original eQUANT eMcFarland results or the original standard inoculum results.

### Reproducibility study

The reproducibility study was performed to demonstrate that the eQUANT system reproducibly prepares a 0.5 McFarland equivalent inoculum, the eMcFarland, at an organism density of 1 – 2e8 ± 0.6 log (2.51 × 10^7^ and 7.96 × 10^8^) CFU/mL from a PBC. The reproducibility of the eQUANT system was assessed across sites (one internal and two external), operators, runs, instruments, and consumable lots. A panel of six organisms was contrived in blood culture bottles with human blood added and incubated on a blood culture monitoring system until positivity. From each positive blood culture, initial eQUANT testing was performed in duplicate by two operators at each site for a total of four eMcFarlands per PBC. The resulting eMcFarlands were plated to confirm colony counts were within the defined range. Due to some *Acinetobacter baumannii* eMcFarland colony counts being below acceptance criteria at one site, additional testing was performed to further evaluate the reproducibility of that organism. Additional testing was performed in triplicate by two operators at one site over 5 days.

## RESULTS

Across three clinical laboratories and Avails Medical, Inc., a total of 578 BC specimens were processed with the eQUANT system for disk diffusion AST. Subsequently, 11 BC specimens were excluded from analysis for the following reasons: three samples exceeded allowed processing time post removal from the eQUANT instrument, two samples ended up being non-target organisms, two samples exceeded the maximum eQUANT run time, one sample was polymicrobial, one positive blood culture bottle used for eQUANT exceeded the 12 hour time limit post positivity, there was a sample mix-up with one sample, and there was insufficient growth for another sample. The 567 organisms present in the study samples, of which 42 were prospective and 525 were contrived samples, are broken down by species as listed in Table 2. As expected, *E. coli* was the most frequently isolated organism from clinical samples (25/42), followed by *K. pneumoniae* (11/42).

**Fig 3:**
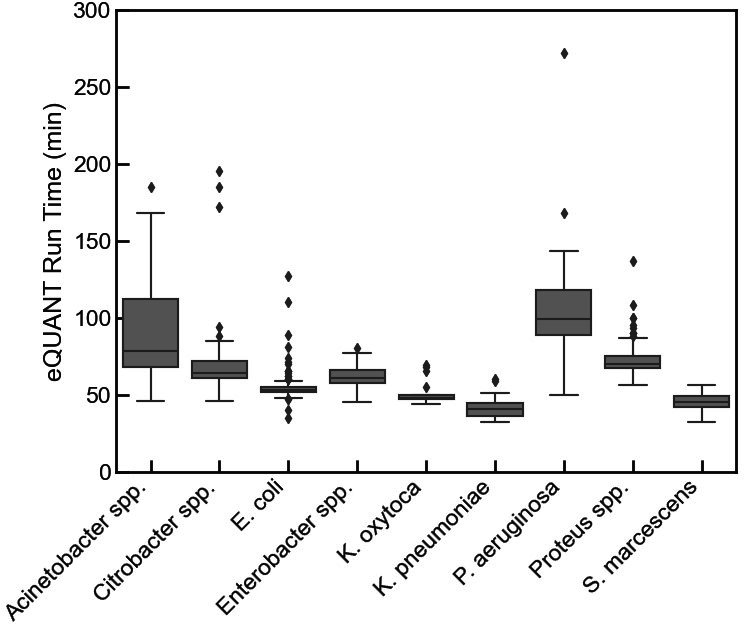
eQUANT run times by species. Box plot showing the distribution of eQUANT run times for different organism groups (*N* = 567). Overall, the eQUANT run times ranged from 32 minutes to 272 minutes with an average of 64.3 minutes (SD = 24.1 minutes). Comparing the different organism groups, *K. pneumoniae* had the fastest average run time of 41.5 minutes and *P. aeruginosa* had the slowest average run time of 105.6 minutes. The center of the box indicates the median, and the bottom and top of the boxes show the 25th and 75th percentiles, respectively. The whiskers extend from the median to 1.5 times the interquartile range, and if the data do not extend to the whiskers, they extend to the minimum and maximum data values. Data points that fall either above or below the whiskers are plotted as diamonds.

The eQUANT run times were captured for all 567 runs, covering nine species or species groups. As shown in Fig. 3, the eQUANT run times ranged from 32 minutes (*S. marcescens*) to 272 minutes (*P. aeruginosa*), with an overall average of 64.3 minutes. Samples that contained Enterobacterales tended to be processed by eQUANT more quickly, in 57 minutes on average, versus samples containing slower-growing organisms like *Acinetobacter* spp. and *P. aeruginosa*. Samples containing *K. pneumoniae* and *S. marcescens* were processed by eQUANT in 41 and 45 minutes on average, respectively. Longest run times were noted for samples containing *P. aeruginosa* and *Acinetobacter* species, with eQUANT run times of 105.6 and 91 minutes on average, respectively.

**Fig 4:**
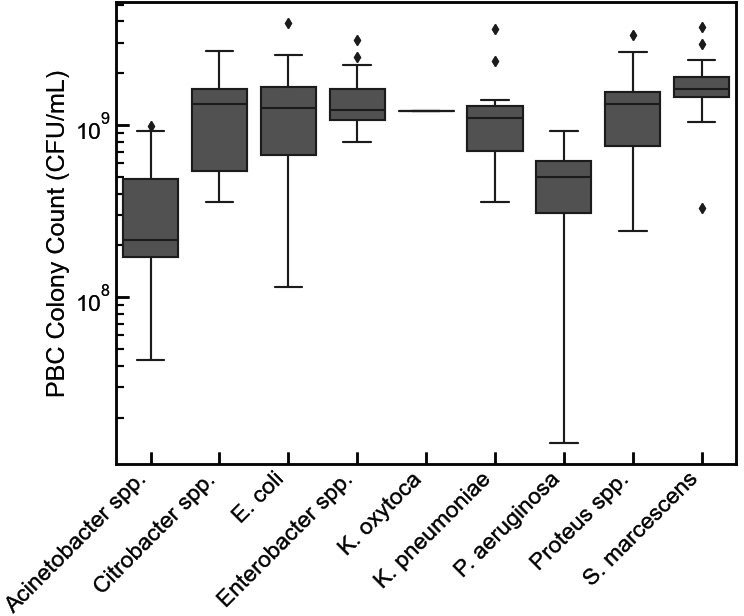
Positive blood culture colony counts. Box plot showing the distribution of colony counts performed on 155 PBC bottles used in the study. Overall, the PBC colony counts ranged from 1.43 × 10^7^ CFU/mL to 3.94 × 10^9^ CFU/mL with *P. aeruginosa* displaying the largest range from 1.43 × 10^7^ CFU/mL to 9.29 × 10^8^ CFU/mL. The center of the box indicates the median, and the bottom and top of the boxes show the 25th and 75th percentiles, respectively. The whiskers extend from the median to 1.5 times the interquartile range, and if the data do not extend to the whiskers, they extend to the minimum and maximum data values. Data points that fall either above or below the whiskers are plotted as diamonds.

Colony counts were prepared on a subset of 115 positive blood culture bottles to evaluate the distribution of organism density for the nine target species or species groups and across three continuous monitoring blood culture systems including the BD Bactec, bioMérieux BacT/Alert 3D, and the BacTAlert Virtuo (Fig. 4). Colony counts ranged from 1.43 × 10^7^ CFU/ mL to 3.94 × 10^9^ CFU/ mL, demonstrating a nearly 300-fold range in bacterial load going into the eQUANT system. The largest range (1.43 × 10^7^ CFU/ mL to 9.29 × 10^8^ CFU/ mL) was detected for *P. aeruginosa*.

**Fig 5:**
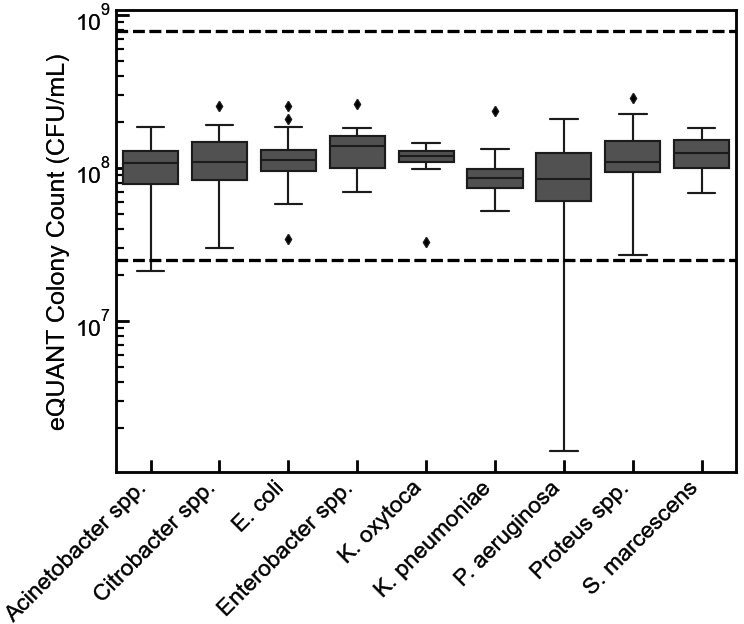
eMcFarland colony counts Box plot showing the distribution of eMcFarland colony counts across the different organism groups represented in the study (*N* = 221). Overall, the eQUANT colony counts ranged from 1.43 × 10^6^ CFU/mL to 2.87 × 10^8^ CFU/mL with an average colony count of 1.14 × 10^8^ CFU/mL. The center of the box indicates the median, and the bottom and top of the boxes show the 25th and 75th percentiles, respectively. The whiskers extend from the median to 1.5 times the interquartile range, and if the data do not extend to the whiskers, they extend to the minimum and maximum data values. Data points that fall either above or below the whiskers are plotted as diamonds.

The eMcFarland colony counts were performed on a subset of 221 eQUANT samples to determine if the eMcFarland suspension density was between 2.51 × 10^7^ and 7.96 × 10^8^ CFU/mL, equivalent to a 0.5 McFarland standard. Of the 221 samples, 219 (99.1%) were within the expected range and therefore met the acceptance criteria for overall samples (Fig. 5). The two samples outside the expected colony count range were one contrived *Acinetobacter* spp. (2.14 × 10^7^ CFU/mL) and one contrived *P. aeruginosa* sample (1.43 × 10^6^ CFU/mL). Even though the inoculum densities of these two samples were below the acceptable count, no errors were observed in downstream disk diffusion AST results.

The results for disk diffusion testing and the performance summary of testing from eQUANT versus standard inocula are shown in Table 3. There are 2,679 pairs of drug-organism results covering the three pathogen groups tested, Enterobacterales, *Acinetobacter spp*., and *P. aeruginosa*. Of these, 1,825 were categorized as susceptible, 169 as intermediate, and 685 as resistant by standard methodology.

Overall, 8 of the 12 antimicrobials tested met the FDA CA acceptance criterion of ≥95%. CA of <95% was found with Enterobacterales for four agents—amoxicillin/clavulanic acid (94%), cefazolin (85%), cefepime (92%), and piperacillin/tazobactam (94%).

For amoxicillin/clavulanic acid, the overall CA of 94% for Enterobacterales was only slightly below the acceptance criterion based on two species. One was *E. coli* with a CA of 94% based on one major and eight minor errors. Only one ME was observed out of 93 susceptible *E. coli* and 150 susceptible Enterobacterales strains. This error was not reproducible in discrepancy testing. Six of the minor errors (75%) were based on a difference in zone sizes of 1 mm–2 mm. The other species was *P. mirabilis* with a CA of 93%, based on three minor errors. All errors were the result of a susceptible comparator result and an intermediate result obtained with the eQUANT eMcFarland inoculum. Amoxicillin/clavulanate, however, met performance criteria with no VME and 0.67% ME.

For cefazolin, the overall CA for all Enterobacterales strains was 85% for the three species evaluated, *E. coli* (82% CA), *P. mirabilis* (85% CA), and *K. pneumoniae* (93%). *K. pneumoniae* did not meet the CA acceptance criterion based on three minor errors, all of which were based on ≤3 mm difference in zone sizes. Cefazolin met performance criteria for VME (0%) and ME (1.44%).

In the case of cefepime, the overall CA for all Enterobacterales evaluated was 92%. Low agreement was observed for cefepime with *Citrobacter freundii* (81%), which resulted from four minor errors, and cefepime with *K. aerogenes* (86%), resulting from three minor errors, with varied differences in zone diameters. Three additional Enterobacterales species did not meet acceptance criteria for CA, including *E. cloacae* (90% CA) based on four minor errors. CA for *K. oxytoca* was 93% based on one minor error where the comparator result was intermediate (20 mm) and the eQUANT result was resistant (18 mm). *P. mirabilis* had a CA of 93% based on one major error (2.86%) and two minor errors. Cefepime, however, met performance criteria for *P. aeruginosa* (95% CA) but did not meet acceptance criteria for ME (9.52%) due to two major errors. The errors were due to a difference in zone sizes of 2 mm–3 mm, as there is no intermediate breakpoint for cefepime/*P. aeruginosa*. Cefepime met performance criteria for VME (0%).

CA of piperacillin/tazobactam with Enterobacterales was only slightly below the acceptance criterion (94%) because of *E. coli*, which only reached a CA of 93% due to 11 minor errors. Piperacillin/tazobactam met CA performance criteria for *Acinetobacter* spp. and *P. aeruginosa*, as well as for VME (0%) and ME (0%).

Results of repeat/discrepancy testing of the 12 organism-drug with VMEs (*n* = 1) and MEs (*n* = 11) are shown in Table S4. None of these errors was reproduced on repeat testing, which showed that standard results were correct in five cases and eQUANT results were correct in four cases, with neither result correct in two cases. Seven of these 12 errors were single discrepancies in a sample and were likely random errors. The remaining errors were three MEs that occurred in a sample containing *Proteus* species which were susceptible to the agents on repeat testing and two MEs that occurred in a sample containing *E. coli*.

In summary, of 2,679 pairs of AST results, error rates were 1 VME for an overall VME rate of 0.15% (1/685) and 11 MEs for an ME rate of 0.6% (11/1,825), meeting overall FDA acceptance criteria. Seven of 12 antimicrobials met all performance requirements for CA, VME, and ME: ampicillin, aztreonam, ceftriaxone, ertapenem, levofloxacin, meropenem, and tobramycin. Taking discrepant results testing for each antimicrobial into consideration, all 12 antimicrobials either met acceptance criteria, or performance was acceptable as VMEs and MEs appeared to be random errors for disk diffusion results obtained with the eQUANT eMcFarland.

The average time from blood culture positivity to the start of disk diffusion AST using the eQUANT eMcFarland was 8.9 hours, compared to 33.4 hours using a standard 0.5 McFarland inoculum prepared from plate, resulting in an average time saving in the disk diffusion AST workflow of 24.5 hours when using the Avails Medical eQUANT system for automated inoculum preparation directly from PBC.

As a component of this study, reproducibility testing was performed (see Materials and Methods section). Overall agreement based on eMcFarland counts across sites, operators, runs, instruments, and consumable lots was 98.9%, demonstrating that the eQUANT system prepares a 0.5 McFarland equivalent inoculum with a high degree of reproducibility (Table 4).

**TABLE 4:** eQUANT reproducibility testing results

Organism	Isolate ID	Agreement
*Escherichia coli*	ATCC 25922	72/72 (100%)
*Pseudomonas aeruginosa*	ATCC 27853	72/72 (100%)
*Klebsiella pneumoniae*	ATCC 700603	72/72 (100%)
*Acinetobacter baumannii*	ATCC 19606	97/102 (95.1%)
*Proteus vulgaris*	ATCC 6380	72/72 (100%)
*Serratia marcescens*	ATCC 14756	72/72 (100%)
Overall agreement		457/462 (98.9%)

## DISCUSSION

Bloodstream infections and sepsis represent major causes of morbidity and mortality worldwide (1, 5). Timely availability of antimicrobial susceptibility testing of the causative bacterial organism is critical for optimal antimicrobial therapy and management of sepsis patients (14). Many recent studies have shown that gram-negative bacteria constitute the majority of bacterial organisms implicated in sepsis (23). Considering the rise in antimicrobial resistance, specifically in GNRs during the past 2 decades, providing AST results faster than with the traditional approach of using bacterial colonies from positive BCs after 24 hour overnight growth on agar plates is essential for optimizing antimicrobial therapy. Timely AST results support antimicrobial stewardship efforts to allow for de-escalation or escalation of antimicrobial therapy, and studies have demonstrated that effective antimicrobial stewardship will lead to significant improvements in management and survival of sepsis patients (24). That said, currently available laboratory tests and systems, such as molecular detection of select antimicrobial resistance (AMR) genes or the use of preliminary AST directly from BC broth, are imperfect for various reasons (10, 25). The detection of select AMR genes may be informative to avoid certain antimicrobial agents, as in the case of treatment for MRSA vs MSSA bacteremia; however, a complete antibiogram is still needed for determining optimal antimicrobial therapy. In addition, the various molecular rapid ID methods (e.g., BioFire, ePlex) offer only a select number of key organisms that are detected from positive BCs, and while such systems have expanded the panels with regard to the number of different organisms that can be detected, there is only a limited number of resistance genes incorporated on these panels. Lastly, such systems are more costly to the laboratory, compared to disk diffusion AST methods. Likewise, while proven somewhat informative over the years of its use, preliminary AST results obtained by disk diffusion testing when inoculating broth from a positive BC bottle directly onto an agar plate have important limitations specifically in the absence of using a standardized organism inoculum size and as to the possibility of having more than one organism present in the BC broth. Both CLSI and EUCAST rapid AST by disk diffusion require some adjustments to the workflow in the clinical laboratory, specifically when testing should be set up at any time of the day when BCs become positive (e.g., evening and overnight shift). In addition, laboratory staff have to be trained and familiar with the alternate breakpoints and modified protocols for evaluation and interpretation of the disk diffusion plates (12, 26, 27).

In our multicenter study, we evaluated the eQUANT system in conjunction with disk diffusion AST in 567 positive blood culture samples. Compared to the standard of care method, which requires a 0.5 McFarland inoculum manually prepared from colonies of an 18–24 hour subcultured positive blood culture, using the eQUANT system allowed us to set up disk diffusion AST for gram-negative bacteria directly from a PBC with an identified target organism. Our study demonstrated that the use of the eMcFarland, generated by the eQUANT system in only 64 minutes on average, produced highly accurate overall disk diffusion AST results (95% CA). These results, which are interpreted in accordance with the FDA guidelines, would be available to clinicians as much as 24 hours sooner than results obtained from the traditional AST approach using the 0.5 McFarland standard from an 18–24 hour bacterial subculture.

In contrast to a standard nephelometer, the eQUANT system quantifies living organisms and is therefore the first of its kind to truly standardize a bacterial inoculum directly from a positive blood culture. It is presently FDA cleared for use with disk diffusion AST for select gram-negative bacteria, identified by rapid ID systems. Currently, the only comparable methods to eQUANT are the direct disk diffusion method per the CLSI M02 and M100 and the EUCAST rapid disk diffusion method for AST directly from positive blood culture bottles, both of which use a non-standardized inoculum, which is droplets directly applied from a positive blood culture (12, 27). The CLSI direct disk diffusion method can only be used for blood cultures up to 8 hours post positivity, whereas the eQUANT system accepts PBCs up to 12 hours post positivity. Both methods, eQUANT and CLSI direct disk diffusion, require rapid organism identification for result interpretation. However, one limitation of the eQUANT system is the need for a rapid ID input to start the run, and the organisms have to match one of the target gram-negative species. In contrast, the CLSI method allows setting up the disk diffusion plate before ID is available and only requires ID information for result interpretation. Another automated sample preparation device for ID of organisms present in PBCs is the Accelerate Arc System, which provides microbial suspensions for organism identification by matrix-assisted laser desorption ionization time-of-flight (MALDI ToF) mass spectrometry; however, this system cannot be used for AST (28). Furthermore, only monomicrobial PBCs should be processed on the eQUANT system. In our study, only 2 out of 567 PBC samples tested (0.35%) could not be processed on the eQUANT system as they were identified as containing non-target organisms. Additionally, only one PBC (0.17%) with a single target species initially detected by rapid identification was polymicrobial on subculture. Besides the Accelerate Pheno BC system, which has polymicrobial identification capability, all other recently cleared direct from PBC AST devices, including the Selux NGP system, the bioMerieux Reveal system, Qlinea ASTar, and Affinity Biosensor’s Lifescale system, have similar limitations as they rely on rapid identification methods for polymicrobial detection and result interpretation (29, 30).

Depending on the organism ID, the CLSI method provides faster (8–10 hours) reading of zone diameters for certain bacteria/antimicrobial combinations. Because the CLSI method uses non-standardized inocula, certain breakpoints had to be adjusted, whereas current FDA breakpoints can be applied using the eQUANT system.

Additional studies with the eQUANT system being used with other automated downstream AST methods such as the MicroScan Walkaway System (Beckman-Coulter, Inc.), the Phoenix system (Becton Dickinson & Co.), or the Vitek system (bioMérieux) may provide additional information regarding the performance of the eQUANT system with several of the automated AST systems that are widely used in the USA, which could have the potential to accelerate the traditional automated blood culture AST workflow by up to 24 hours, enabling same-day AST results. A pilot study of the eQUANT system showed categorical agreement of 97.5%, 96.9%, and 96.7% by disk diffusion, MicroScan, and Vitek methods, respectively (16).

Lastly, additional clinical studies would be needed to better describe the true clinical impact and the effects on the management of sepsis patients when using the eQUANT system in real-time for BCs from sepsis patients.

## SUPPLEMENTAL MATERIAL

Supplemental tables jcm.01606-24-s0001.docx Tables S1 to S4.

ASM does not own the copyrights to Supplemental Material that may be linked to, or accessed through, an article. The authors have granted ASM a non-exclusive, world-wide license to publish the Supplemental Material files. Please contact the corresponding author directly for reuse.
