# Supplementary material for: Multicenter evaluation of the eQUANT system for use with disk diffusion AST of gram-negative bacteria directly from positive blood cultures
Source: J Clin Microbiol. 2025 May 9;63(6):e01606-24. doi: 10.1128/jcm.01606-24 (PMC12153300; doi:10.1128/jcm.01606-24)
Supplement: Supplemental tables — Tables S1 to S4. [file jcm.01606-24-s0001.docx]

**Supplementary Table 1: Resistance mechanisms of 95 challenge isolates with genotypically identified resistance markers used in the study (1)**

| **Bacterial Organism** | **Resistance Mechanisms** |
| --- | --- |
| *Acinetobacter baumannii* | aac(3)-Ia, aadA1, ABAF, ADC-33, ADEC, ant(3")-IIa, aph(6)-Id, armA, mph(E), msr(E), OXA-23, OXA-82, qacEdelta1, strA, sul1, tet(B) |
| *Acinetobacter baumannii* | aac(6')-Ib-AKT, aadA1, ABAF, ADC-162, ADEC, aph(3')-Ia, aph(6)-Id, aphA6, armA, catB8, mph(E), msr(E), OXA-23, OXA-66, qacEdelta1, strA, sul1, sul2, tet(B) |
| *Acinetobacter baumannii* | aac(3)-Ia, aacA16, ABAF, ADC-30, ADEC, aph(6)-Id, OXA-66, OXA-72, strA, sul2, tet(B) |
| *Acinetobacter baumannii* | aac(3)-IIe, aac(6')-Ian, ABAF, ADC-214, ant(3")-IIa, aph(6)-Id, OXA-24, OXA-65, strA, sul2, TEM-1 |
| *Acinetobacter baumannii* | aac(3)-Ia, aadA1, ABAF, ADC-30, ADEC, aph (3')-la, aph(6)-ld, OXA-66, OXA-77, qacEdelta1, strA, sul1, sul2, TEM-1D, TEM(B) |
| *Acinetobacter baumannii* | ABAF, ADC-79, ant(3")-IIa, OXA-100, OXA-58, sul2 |
| *Acinetobacter baumannii* | ABAF, ADC-162, ADEC, aph(6)-Id, mph(E), msr(E), OXA-23, OXA-66, strA, sul2, tet(B) |
| *Acinetobacter baumannii* | aac(3)-IIe, aac(6')-Ian, ABAF, ADC-214, ant(3")-IIa, aph(6)-Id, OXA-24, OXA-65, strA, sul2, TEM-1 |
| *Acinetobacter baumannii* | ABAF, ADC-30, ADEC, aph(3')-Ia, aph(6)-Id, mph(E), msr(E), OXA-66, OXA-72, strA, sul2, TEM-1D, tet(B) |
| *Citrobacter freundii* | CMY-108, QnrB13 |
| *Citrobacter freundii* | aac(3)-IId, aac(6')-Ib-cr, aadA1, ARR-3, catB3, CMY-48, dfrA1, mph(A), OXA-1, OXA-48, qacEdelta1, QnrB19, sat2, sul1, sul2, TEM-1, tet(D) |
| *Enterobacter cloacae* | aadA1, aadA2, sul1 |
| *Enterobacter cloacae* | aac(3)-IId, aac(6')-Ib, aadA1, aadA2, ACT-17, armA, ble-MBL, catB4, CTX-M-15, dfrA12, floR2, fosA, LAP-2, mph(E), msr(E), NDM-1, Omp36, OmpC, OmpC2, OmpK36, OXA-1, OXA-9, qacEdelta1, QnrS1, rmtB4, sul1, TEM-1, tet(G) |
| *Enterobacter cloacae* | aac(6')-Ib-cr, ACT-16, aph(3")-Ib, aph(6)-Id, ble-MBL, catB4, CTX-M-15, dfrA14, floR, NDM-1, OXA-1, QnrB1, rmtC, sul1, sul2, TEM-191, tet(A), tet® |
| *Enterobacter cloacae* | aac(6')-Ib, aadA1, ACT-45, aph(3")-Ib, aph(3')-Ia, aph(6)-Id, dfrA14, KPC-3, OmpF2, OXA-9, sul2, TEM-1A |
| *Enterobacter cloacae* | aac(6')-IIc, aadA2, aadB, ACT-7, dfrA1, dfrA12, dfrA18, mph(A), QnrB2, strA, strB, sul1, sul2, TEM-1B, tet(D), VIM-1 |
| *Enterobacter cloacae* | aac(3)-IIa, ACT-7, CTX-M-15, dfrA14, KPC-2, OXA-1, strA, strB, sul2, TEM-1B |
| *Enterobacter cloacae* | aac(6')-Ib, aadA1, ACT-45, aph(3")-Ib, aph(3')-Ia, aph(6)-Id, dfrA14, KPC-3, OXA-9, SHV-12, sul2, TEM-1A |
| *Enterobacter cloacae* | aac(6')-IIc, aadA2, ACT-16, aph(6)-Id, armA, CTX-M-15, dfrA12, ere(A), mph(E), msr(E), strA, sul1, sul2, TEM-1B |
| *Enterobacter cloacae* | aac(6')-Ib, aph(3')-Ia, aph(6)-Id, dfrB5, NDM-1, SHV-12, strA, TEM-1B |
| *Enterobacter cloacae* | aac(6')-Ib, aadA1, dfrA14, KPC-3, OmpF2, OXA-9, SHV-12, strA, strB, sul2, TEM-1A |
| *Enterobacter cloacae* | aadA2, ACT-16, aph(6)-Id, dfrA12, KPC-2, LAP-2, mph(A), QnrS1, strA, sul1, TEM-1B, tet(A), tet(R) |
| *Enterobacter cloacae* | aac(6')-Ib, aadA1, aph(3')-Ib, aph(6)-Id, dfrA14, KPC-3, OmpF2, OXA-9, sul2, TEM-1A |
| *Escherichia coli* | aac(3)-Ib, aac(6')-Ib-cr5, ACT-16, aph(3')-Ia, ARR-3, catB3, KPC-6, mph(A), OXA-1, qacEdelta1, qacF, sul1, TEM-1A |
| *Escherichia coli* | aac(6')-Ib-cr, aadA5, ACRF, catB4, dfrA17, KPC-3, MDF(A), mph(A), OXA-1, sul1, tet(A), tet(R) |
| *Escherichia coli* | ACRF, EMRD, MDF(A) |
| *Escherichia coli* | ACRF, MDF(A) |
| *Escherichia coli* | ACRF, EC-19, EMRD |
| *Escherichia coli* | aadA5, ACRF, dfrA17, qacEdelta1, sul1, TEM-12 |
| *Escherichia coli* | mcr-1 |
| *Escherichia coli* | aadA5, aph(3')-Ib, aph(6)-Id, dfrA17, mph(A), QnrB19, sul1, sul2, TEM-1B, tet(A) |
| *Escherichia coli* | aph(3')-Ib, aph(6)-Id, dfrA8, QnrB19, sul2, TEM-1A, tet(B) |
| *Escherichia coli* | aadA5, AmpC1_Ecoli, aph(3')-Ib, aph(6)-Id, dfrA17, mph(A), QnrB19, sul1, sul2, TEM-1B, tet(A) |
| *Escherichia coli* | aadA2, QnrB2, SHV-105, sul1, TEM-1B |
| *Escherichia coli* | TEM E104K, TEM G238S |
| *Escherichia coli* | CMY-2/FOX, CTX-M-1 group, TEM |
| *Escherichia coli* | CMY-2/FOX, CTX-M-1 group, NDM, SHV E240K, SHV G238S |
| *Escherichia coli* | CMY-2/FOX, NDM, SHV E240K, SHV G238S- Positive |
| *Escherichia coli* | CMY-2/FOX, CTX-M-1 group, NDM, TEM |
| *Escherichia coli* | CMY-2/FOX, CTX-M-1 group, NDM, TEM |
| *Escherichia coli* | CMY-2/FOX, CTX-M-1 group, NDM, TEM |
| *Escherichia coli* | CMY-2/FOX, CTX-M-1 group, NDM, TEM |
| *Escherichia coli* | NDM, TEM E104K |
| *Escherichia coli* | KPC, TEM |
| *Escherichia coli* | CTX-M-1 group, KPC, TEM |
| *Escherichia coli* | CTX-M-1 group, NDM, TEM |
| *Escherichia coli* | aac(6')-IB3, aadA2, ACRF, aph(3')-Ia, aph(6)-Id, ble-MBL, catA1, CMY-6, CTX-M-15, dfrA12, dfrA29, dfrA4, EMRD, NDM-1, OmpF, OXA-2, qacEdelta1, rmtC, strA, sul1, TEM-1, tet(B) |
| *Escherichia coli* | aac(3)-IIa, aac(6')-Ib-D181Y, aadA5, ACRF, ble-MBL, catB4, CMY-6, dfrA17, mph(A), NDM-1, OXA-1, qacEdelta1, rmtC, sul1, tet(A), tet® |
| *Escherichia coli* | aac(3)-IIa, aadA1, aadA5, CMY-42, CTX-M-15, dfrA17, mph(A), NDM-6, OXA-1, OXA-9, QnrS1, sul1, TEM-1A, tet(B) |
| *Escherichia coli* | aac(6')-IB3, ACRF, aph(3")-Ib, ble-MBL, CMY-6, dfrA8, EMRD, NDM-1, qacEdelta1, sul1, sul2, TEM-1, tet(A), tet® |
| *Escherichia coli* | aadA1, ACRF, catA1, CMY-42, dfrA1, EMRD, OXA-1, sat2, tet(B) |
| *Escherichia coli* | CMY-42, dfrA1, OXA-1, sat-2A, tet(B) |
| *Escherichia coli* | catB4, CTX-M-15, dfrA8, OXA-1, tet(A), tet(R) |
| *Escherichia coli* | CMY-2 |
| *Escherichia coli* | aac(6')-Ib-AKT, aadA5, ACRF, catB4, CTX-M-15, dfrA17, EMRD, KPC-3, MDF(A), mph(A), OXA-1, sul1, TEM-1B, tet(A), tet(R) |
| *Escherichia coli* | aac(3)-IId, aph(3")-Ib, aph(6)-Id, dfrA17, EC-5, EMRD, KPC-3, MDF(A), sul2, TEM-1, tet(B) |
| *Escherichia coli* | aac(3)-IIa, aac(6')-Ib-cr, aadA5, catB4, CTX-M-15, dfrA1, dfrA17, DHA-1, mph(A), OXA-1, QnrB4, sul1, tet(B) |
| *Escherichia coli* | aadA5, CMY-42, CTX-M-15, dfrA17, mph(A), sul1, TEM-1B, tet(B) |
| *Escherichia coli* | NDM-5, OXA-1, strA, strB, catB3, floR, mcr-1, aac(6')-Ib-cr, arr-3), sul1, sul2, tet(A) |
| *Escherichia coli* | ACRF, EMRD, OmpF, TEM-1B |
| *Klebsiella aerogenes* | OXA-48 |
| *Klebsiella aerogenes* | aph(6)-Id, dfrA14, sul2 |
| *Klebsiella aerogenes* | CMY2-MIR-ACT-EC, Omp35, Omp36, OmpK35, OmpK36 |
| *Klebsiella aerogenes* | aac(3)-IId, catB3, IMP-4, OXA-1, SFO-1, strA, strB, sul1, TEM-1B |
| *Klebsiella pneumoniae* | aac(6')-Ib, aadA1, aph(3')-Ib, aph(6)-Id, dfrA14, EMRD, KDEA, KPC-3, Omp35, OmpK35, oqxA, oqxB, OXA-9, SHV-12, sul2, TEM-1A |
| *Klebsiella pneumoniae* | KPC-3, OmpK35, oqxA, oqxB, SHV-11, TEM-1B |
| *Klebsiella pneumoniae* | aac(3)-IId, aac(6')-Ib-cr, aadA1, aadA2, aph(3")-Ib, aph(6)-Id, ARR-2, catA1, CTX-M-15, dfrA12, dfrA14, EMRD, ere(A), fosA5, KDEA, Omp35, OmpK35, oqxB9, oqxB9, OXA-181, qacEdelta1, SHV-26, sul1, sul2, tet(A), tet® |
| *Klebsiella pneumoniae* | aac(3)-IIa, aac(6')-Ib-cr, aac(6')-Il, aph(3')-Ia, aph(6)-Id, catB4, CTX-M-15, dfrA1, EMRD, KDEA, mph(A), Omp35, OmpK35, oqxA, oqxB25, OXA-1, qacEdelta1, SHV-11, strA, sul1, tet(A), tet(R), VIM-27 |
| *Klebsiella pneumoniae* | aac(6')-IIa, aadA1, aph(3")-Ib, aph(3')-VI, aph(6)-Id, armA, ARR-2, ble-MBL, catB11, cmlA5, CMY-4, CTX-M-15, dfrA1, EMRD, fosA5, KDEA, mph(E), msr(E), NDM-1, oqxA, oqxB, OXA-10, qacEdelta1, SHV-11, sul1, sul2, tet(A), tet® |
| *Klebsiella pneumoniae* | aac(3)-IId, aac(6')-Ib, aadA1, catA1, catB4, CTX-M-15, dfrA14, EMRD, floR, fosA5, KDEA, Omp35, OmpK35, oqxA, oqxB25, OXA-1, OXA-9, SHV-11, sul2, TEM-1A |
| *Klebsiella pneumoniae* | aac(3)-Iva, aac(6')-Ib, aadA1, aph(4)-Ia, catA1, cmlA1, dfrA12, EMRD, KDEA, Omp35, OmpK35, oqxA, oqxB, SHV-12, sul1, sul3 |
| *Klebsiella pneumoniae* | aac(6')-Ib, aadA2, catA1, dfrA12, EMRD, KDEA, mph(A), Omp35, OmpK35, oqxA, oqxB, qacEdelta1, sul1, TEM-1A, tet(D) |
| *Klebsiella pneumoniae* | aac(3)-IIa, aac(6')-Ib-D181Y, aadA2, aph(3')-Ib, aph(6)-Id, ble-MBL, catB4, CMY-6, CTX-M-15, dfrA14, EMRD, KDEA, mph(A), NDM-1, Omp35, OmpK35, oqxA, oqxB, OXA-1, qacEdelta1, QnrB9, rmtC, SHV-53, sul1, sul2, TEM-1, tet(A), tet® |
| *Klebsiella pneumoniae* | aac(3)-IId, aac(6')-Ib, aadA1, aph(3")-Ib, aph(6)-Id, ARR-2, catA1, catB4, cmlA5, CTX-M-15, dfrA1, EMRD, ere(A), fosA5, KDEA, Omp35, OmpK35, oqxA, oqxB20, OXA-1, OXA-232, OXA-9, qacEdelta1, sat2, SHV-11, sul1, sul2, TEM-1A |
| *Klebsiella pneumoniae* | aac(3)-IId, aac(6')-Ib, aadA1, aadA2, aph(3")-Ib, aph(3')-VI, aph(6)-Id, armA, ble-MBL, catA1, catB4, CTX-M-15, dfrA1, dfrA12, EMRD, fosA5, KDEA, mph(E), msr(E), NDM-1, oqxA, oqxB20, OXA-1, OXA-232, OXA-9, qacEdelta1, sat2, SHV-11, sul1, sul2, TEM-1A |
| *Klebsiella pneumoniae* | aac(3)-IId, aadA2, aph(3")-Ib, aph(6)-Id, CTX-M-14, dfrA12, DHA-1, EMRD, erm(42), floR2, KDEA, Omp35, Omp36, OmpC, OmpC2, OmpK35, OmpK36, oqxA, oqxB, qacEdelta1, rmtB1, SHV-11, sul2, TEM-1, tet(G) |
| *Pseudomonas aeruginosa* | aac(3)-Id, aac(6')-Il, aadA2, aph(3')-IIb, bcr1, cmlA6, dfrB5, floR2, fosA, mexA, mexE, mexX, OXA-4, OXA-486, PDC-3, qacEdelta1, qacF, tet(G), VIM-2 |
| *Pseudomonas aeruginosa* | ant(2")-la, aph(3')-IIb, aph(6)-Id, bcr1, bcr1, bcr1, catB7, fosA, mexA, mexE, mexX, OXA-396, PDC-3, sul1, sul1, sul1, tet(A), tet(R), tet(R), tet(R), VIM-4 |
| *Pseudomonas aeruginosa* | aac(6')-33, ant(2")-la, aph(3')-IIb, bcr1, catB7, fosA, KPC-5, mexA, mexE, mexX, mph(E), msr(E), OXA-50, PDC-108, qacEdelta1, sul1 |
| *Pseudomonas aeruginosa* | aac(3)-Id, aac(6')-Il, aadA2, aph(3')-IIb, bcr1, cmlA6, dfrB5, floR2, fosA, mexA, mexE, mexX, OXA-4, OXA-486, PDC-3, sul1, tet(G), VIM-2 |
| *Pseudomonas aeruginosa* | aac(6')-IIc, aph(3')-IIb, bcr1, fosA, KPC-5, mexA, mexE, mexX, OXA-2, OXA-50, PDC-103 |
| *Pseudomonas aeruginosa* | aac(6')-IIc, aadA7, aph(3')-IIb, catB7, cmlB, IMP-1, mexA, mexE, OXA-101, OXA-395, OXA-9, PDC-3, sul1 |
| *Pseudomonas aeruginosa* | aac(3)-Id, aac(6')-Il, aadA2, aph(3')-IIb, bcr1, cmlA6, dfrB5, floR2, fosA, mexA, mexE, mexX, OXA-4, OXA-486, PDC-3, sul1, tet(G), VIM-2 |
| *Pseudomonas aeruginosa* | aph(3')-IIb, bcr1, catB7, fosA, mexA, mexE, mexX, OXA-494, PDC-15 |
| *Pseudomonas aeruginosa* | aph(3')-IIb, bcr1, catB7, mexA, mexE, mexX, OXA-50, PDC-173 |
| *Pseudomonas aeruginosa* | bcr1 |
| *Pseudomonas aeruginosa* | ant(2")-la, aph(3')-IIb, bcr1, catB7, fosA, mexA, mexE, mexX, OXA-50, PAO, sul1 |
| *Pseudomonas aeruginosa* | bcr1, OXA-50 |
| *Pseudomonas aeruginosa* | aph(3')-IIb, bcr1, catB7, fosA, mexA, mexE, mexX, OXA-395, PDC-5 |
| *Proteus mirabilis* | cmlA1, dfrA1, dfrA14, KPC-2, OXA-10, sul1, sul2, tet (D), tet(J) |
| *Proteus mirabilis* | aac(6')-Ib, dfrA1, KPC-3, qacEdelta1, QnrB19, sat2, sul1, TEM-1A, tet(J) |
| *Proteus mirabilis* | qacEdelta1, sul1, tet(J) |
| *Serratia marcescens* | ant(2")-la, catB4, FOX-5, mph(E), msr(E), OXA-2, QnrA1, sul1 |
| *Serratia marcescens* | ant(2")-la, aphA16, SHV-105, SRT-2 |
| *Serratia marcescens* | aph(3')-Ib, aph(6)-Id, armA, ARR-2, cmlA5, CMY-16, dfrA12, dfrA14, OXA-10, sul1, sul2, tet(A), tet(R) |

**Supplementary Table 2: FDA STIC and CLSI M100 Zone Diameter AST Breakpoints (mm) used in the current study**

| **Antimicrobial Agent** | **Organism Group** | **S** | **I** | **R** | **Source** |
| --- | --- | --- | --- | --- | --- |
| Amoxicillin/Clavulanate | *Enterobacterales* | ≥18 | 14-17 | ≤13 | STIC/M100 |
| Ampicillin | *Enterobacterales* | ≥17 | 14-16 | ≤13 | STIC/M100 |
| Aztreonam | *Enterobacterales* | ≥21 | 18-20 | ≤17 | STIC/M100 |
|  | *P. aeruginosa* | ≥22 | 16-21 | ≤15 | STIC/M100 |
| Cefazolin | *Enterobacterales* | ≥23 | 20-22 | ≤19 | STIC/M100 |
| Cefepime | *Enterobacterales* | ≥25 | 19-24 | ≤18 | STIC/M100 |
|  | *P. aeruginosa* | ≥18 | - | ≤17 | STIC |
| Ceftriaxone | *Enterobacterales* | ≥23 | 20-22 | ≤19 | STIC/M100 |
| Ertapenem | *Enterobacterales* | ≥22 | 19-21 | ≤18 | STIC/M100 |
| Gentamicin | *Enterobacterales* | ≥15 | 13-14 | ≤12 | STIC |
|  | *P. aeruginosa* | ≥15 | 13-14 | ≤12 | STIC/M100 |
| Levofloxacin | *Enterobacterales* | ≥21 | 17-20 | ≤16 | STIC/M100 |
|  | *P. aeruginosa* | ≥22 | 15-21 | ≤14 | STIC/M100 |
| Meropenem | *Enterobacterales* | ≥23 | 20-22 | ≤19 | STIC/M100 |
|  | *P. aeruginosa* | ≥19 | 16-18 | ≤15 | STIC/M100 |
|  | *Acinetobacter* spp. | ≥18 | 15-17 | ≤14 | STIC/M100 |
| Piperacillin/Tazobactam | *Enterobacterales* | ≥25 | 21-24 | ≤20 | STIC/M100 |
|  | *P. aeruginosa* | ≥23 | 19-22 | ≤18 | STIC |
|  | *Acinetobacter* spp. | ≥21 | 18-20 | ≤17 | STIC/M100 |
| Tobramycin | *Enterobacterales* | ≥15 | 13-14 | ≤12 | STIC |
|  | *P. aeruginosa* | ≥15 | 13-14 | ≤12 | STIC |

**Supplementary Table 3:**

**CLSI M100 Zone Diameter AST breakpoints (mm) that differ from FDA STIC breakpoints shown in Supplementary Table 2.**

| **Antimicrobial Agent** | **Organism Group** | **S** | **I** | **R** | **Source** |
| --- | --- | --- | --- | --- | --- |
| Cefepime | *P. aeruginosa* | ≥18 | 15-17 | ≤14 | M100 |
| Gentamicin | *Enterobacterales* | ≥18 | 15-17 | ≤14 | M100 |
| Piperacillin/Tazobactam | *P. aeruginosa* | ≥22 | 18-21 | ≤17 | M100 |
| Tobramycin | *Enterobacterales* | ≥17 | 13-16 | ≤12 | M100 |
|  | *P. aeruginosa* | ≥19 | 13-18 | ≤12 | M100 |

**Supplementary Table 4: Discrepancy testing results**

| **Sample**  **ID** | **DD ABx** | **Organism** | **Sample Type** | **Orig. Std**  **Zone** | **Orig. Std**  **Int** | **Orig. EQ**  **Zone** | **Orig. EQ**  **Int** | **Error** | **RepeatDisk. Std**  **Zone** | **Repeat Disk.**  **Std Int** | **Repeat Disk. EQ**  **Zone** | **Repeat Disk.**  **EQ Int** | **Error** | **Error**  **Reproduced** | **Correct original result** |
| --- | --- | --- | --- | --- | --- | --- | --- | --- | --- | --- | --- | --- | --- | --- | --- |
| 1021 | Meropenem | *Serratia marcescens* | Stock | 23 | S | 18 | R | ME | 18 | R | 18 | R | n/a | No | EQ |
| 1030 | Cefazolin | *Proteus spp* | Stock | 34 | S | 6 | R | ME | 21 | I | 21 | I | n/a | No | neither |
| 1075 | Meropenem | *Escherichia coli* | Stock | 23 | S | 18 | R | ME | 19 | R | 18 | R | n/a | No | EQ |
| 2034 | Cefepime | *Pseudomonas*  *aeruginosa* | Stock | 18 | S | 16 | R | ME | 18 | S | 18 | S | n/a | No | STD |
| 2037 | Cefepime | *Pseudomonas*  *aeruginosa* | Stock | 18 | S | 15 | R | ME | 21 | S | 22 | S | n/a | No | STD |
| 2044 | Aztreonam | *Proteus spp* | Stock | 38 | S | 6 | R | ME | 39 | S | 33 | S | n/a | No | STD |
| 2044 | Cefepime | *Proteus spp* | Stock | 34 | S | 17 | R | ME | 36 | S | 31 | S | n/a | No | STD |
| 2044 | Ceftriaxone | *Proteus spp* | Stock | 35 | S | 19 | R | ME | 42 | S | 38 | S | n/a | No | STD |
| 3007 | Ceftriaxone | *Enterobacter spp* | Prospective | 24 | S | 15 | R | ME | 14 | R | 14 | R | n/a | No | EQ |
| 3011 | Gentamicin | *Escherichia coli* | Prospective | 26 | S | 7 | R | ME | 6 | R | 7 | R | n/a | No | EQ |
| 3012 | Gentamicin | *Escherichia coli* | Prospective | 7 | R | 23 | S | VME | 23 | S | 23 | S | n/a | No | EQ |
| 3012 | Amoxicillin/  Clavulanate | *Escherichia coli* | Prospective | 18 | S | 13 | R | ME | 15 | I | 15 | I | n/a | No | neither |

**Reference:**

1. Centers for Disease Control and Prevention. CDC & FDA Antimicrobial Resistance Isolate Bank. Available at:

<https://www.cdc.giv/ARIsolateBank/Search> . Last accessed: February 22, 2025
